# The Role of Different Subsets of Regulatory T Cells in Immunopathogenesis of Rheumatoid Arthritis

**DOI:** 10.1155/2012/805875

**Published:** 2012-10-24

**Authors:** Maryam Gol-Ara, Farhad Jadidi-Niaragh, Reza Sadria, Gholamreza Azizi, Abbas Mirshafiey

**Affiliations:** ^1^Department of Immunology, School of Public Health, Tehran University of Medical Sciences, P.O. Box 6446, Tehran 14155, Iran; ^2^Imam Hassan Mojtaba Hospital, Alborz University of Medical Sciences, Karaj, Iran

## Abstract

Rheumatoid arthritis (RA) is a common autoimmune disease and a systemic inflammatory disease which is characterized by chronic joint inflammation and variable degrees of bone and cartilage erosion and hyperplasia of synovial tissues. Considering the role of autoreactive T cells (particularly Th1 and Th17 cells) in pathophysiology of RA, it might be assumed that the regulatory T cells (Tregs) will be able to control the initiation and progression of disease. The frequency, function, and properties of various subsets of Tregs including natural Tregs (nTregs), IL-10-producing type 1 Tregs (Tr1 cells), TGF-**β**-producing Th3 cells, CD8^+^ Tregs, and NKT regulatory cells have been investigated in various studies associated with RA and collagen-induced arthritis (CIA) as experimental model of this disease. In this paper, we intend to submit the comprehensive information about the immunobiology of various subsets of Tregs and their roles and function in immunopathophysiology of RA and its animal model, CIA.

## 1. Introduction

Identification of regulatory T cells (Tregs) led to breaking the dichotomy of Th1/Th2 responses in several pathogenic and autoimmune conditions. It is shown that Th1 cells have the deleterious function in immunopathogenesis of several autoimmune diseases, whereas Th2 cells exert favorable responses [1, 2]. Tregs suppress several autoreactive responses and maintain self-tolerance in immune system. Tregs have recently received an increased interest in the past decade, placing them at the centre of immuno-suppressive reactions, since understanding the development and functions of immunoregulatory cells, the majority of which are CD4^+^ T cells, may elucidate the etiology for loss of self-tolerance [3, 4]. Since the main feature of several autoimmune diseases such as rheumatoid arthritis (RA) is the inflammation and autoreactive responses, identifying the precise function of these cells in disease course can help us to design the new therapeutic methods for treatment of RA in the future.

## 2. Regulatory T Cells

The term of immunosuppression was described in 1970s by Gershon and Kondo for the first time [5]. In turn, Sakaguchi et al. introduce the suppressor or Tregs with increased CD25 expression [6]. Since then, various studies tried to find the different features and functions of these cells in immune system and the immunopathophysiology of different diseases such as cancer and autoimmune diseases. Cancer is usually associated with increased immunosuppression, whereas in autoimmune diseases, immunosuppression is usually impaired. In addition, maintaining the self-tolerance in prevention of autoimmune responses is very important, which is impaired in autoimmune diseases. Thus, Tregs are the deleterious cells in pathogenesis of cancer, but protective cells in autoimmune diseases. Moreover, in infectious diseases Tregs with limiting the inflammatory responses (specially in chronic infections) decrease the tissue damage [7]. It is showed that Tregs can suppress immune responses via various mechanisms including cell-contact-dependent and independent ones [8]. It is demonstrated that nTregs exert their suppressive effects obviously through cell-contact-dependent mechanisms by membrane-bound molecules whereas inducible Tregs basically use the contact-independent mechanisms which are based mainly on cytokines such as IL-10 and TGF-*β* [9]. Although Tregs are usually hyporesponsive to antigenic stimulation or polyclonal activation *in vitro*, it is reported that they can proliferate *in vivo* [10, 11]. Thus, conditional microenvironment that Tregs encircled in that is very important factor in function and behavior of Tregs.

It is now known that various subsets of Tregs exist in immune system including nTregs, CD8^+^ Treg, Tr1 regulatory cells, Th3 cells, and natural killer like T (NKT) cells. In other classification, and Tregs are divided into two subgroups, natural Tregs (nTregs) and inducible Tregs (iTregs). nTregs develop in the thymus during the selection process whereas iTregs develop in the periphery from naive (or in some condition from differentiated) T cells following antigenic stimulation in semispecific conditions [12]. Like the other conventional subsets of T cells, Tregs also experience the selection process in thymus. Their selection is based on the TCR recognition affinity of self-antigens-presented by antigen-presenting cells (APCs). High affinity recognition leads to negative selection of thymocytes and clonal deletion via apoptosis whereas weak affinity recognition of self-antigens leads to positive selection of these thymocytes and survival of them. It seems that Tregs also experience the positive selection in the thymus, but the affinity strength of them for recognition of self-antigens on APCs is between that required for the positive and negative selection for other conventional T cells [13, 14]. On the other hand, it is suggested that commitment stage of thymocytes to Treg lineage may be prior to selection process via TCR recognition affinity and weak TCR affinity recognition is sufficient for survival of Tregs, probably [15]. This fact that TCR of Tregs is more sensitive (10- to 100-fold) to antigenic stimulation than conventional T cells further substantiates this claim [16]. 

## 3. Natural Regulatory T Cells

A subtype of CD4^+^ T cells that develop in the thymus and can constitutively express the high levels of CD25 (IL-2R*α*) are named nTregs. nTregs approximately constitute about 5 to 10% of peripheral CD4^+^ T cells in both human and mice [17]. It is demonstrated that nTreg migration from thymus to periphery in day 3 of mice life is working [18]. Although, identification of CD25 as a nTreg marker could help us in more recognition of these cells, but we now know that activated T cells can also express the high levels of this molecule on their surface whereas it has been reported that even the highest CD25-expressing T cells contain about 50% of recently activated T cells [19]. Thus, CD25 expression may be not the trustable marker for discrimination of nTregs from conventional T cells and particularly from activated T cells. Moreover, it is reported that only 2–4% of cells with the highest CD25 expression can be considered regulatory [20]. Hence, some molecules are also suggested as a discrimination marker for nTregs such as CTLA-4, L-selectin (CD62L), glucocorticoid-induced TNFR (GITR) family-related protein, OX40 (CD134), folate receptor 4 (FR4), neuropilin-1, and CD103 [21]. However, expression of these molecules is not limited to nTregs, and other subsets of T cells can also express these molecules in some condition. In addition, nTregs cannot express constitutively these molecules on their surface.

Identification of forkhead box protein P3 (FoxP3) as a Treg-specific transcription factor from X-linked forkhead family in 2003 leads to more success in discrimination of nTregs from other CD4^+^ T cells [22]. It is suggested that impaired or decreased expression of FoxP3 in nTregs can lead to impaired nTreg suppressing function [8]. Forced expression of FoxP3 in conventional T cells via retroviral transduction leads to acquiring the regulatory function in these cells that further substantiate the role of FoxP3 in regulatory function of nTregs [23]. It has been also shown that FoxP3 expression is critical for development and maintenance of nTregs in thymus and periphery [24]. Additionally, it has been shown that FoxP3 has an important role in the peripheral maintenance of nTreg phenotype stability, such as anergy and IL-2 dependence [25]. In the molecular level, it is showed that FoxP3 function can repress nuclear factor of activated T cells-activation protein 1-(NFAT-AP1-) dependent transcription and formation of FoxP3-NFAT complex that suppresses IL-2 expression and confers an immunosuppressive phenotype [26]. It has also become apparent that TGF-*β* has an important role in maintaining of FoxP3 expression and hence immunosuppressive function of nTregs [27]. It should be noted that TGF-*β* signaling in peripheral nTregs is pivotal for immunosuppressive effects of them on CD8^+^ T cells, Th1 cells, and NK cells [28]. Although, TGF-*β* mRNA is not increased in nTregs, it has been reported that membrane-bound form of TGF-*β* is elevated on the surface of nTregs and is critical for their action [29]. In contrast, it is reported that nTreg development is intact in TGF-*β* receptor dominant negative mice [30]. On the other hand, it is showed that TNF-*α*  can inhibit FoxP3 expression in nTregs [31]. Additionally, it is noted that IL-2 also plays a critical role in peripheral maintenance of nTregs [32]. Based on this role of IL-2 in maintenance of nTregs, it is proposed that consumption of IL-2 by nTregs are one immunosuppressive mechanism of these cells through which, they can deprive other conventional non-Treg cells from IL-2 [33]. 

FoxP3 is a bona fide marker for nTreg? [34]. In spite of the fact that FoxP3 expression is critical for nTreg function, it has been detected that its expression is not confined to Tregs and significant numbers of activated T cells can also express FoxP3 [35]. Moreover, it is demonstrated that, FoxP3 expression in activated T cells may lead to acquiring the suppressive function or not [36]. It has been also shown that upregulation of FoxP3 in activated T cells is controlled by signal transducer and activator of transcription 5- (STAT5) dependent manner. Thus, cytokines such as IL-2, IL-7, and IL-15 that activate STAT5 manage FoxP3 expression in activated cells as well as nTregs [37]. Roncarlo and Gregori suggest that depending on the cell subset and/or the stage of T-cell differentiation, FoxP3 expression can exert different functions and act as a negative or positive regulator. This is consistent with the fact that FoxP3 is a gene highly subjected to epigenetic modifications, which may contribute to diversifying its function [34]. Interestingly, it is suggested that activated FoxP3^+^ non-Treg cells may be a reservoir of silent Tregs that regain their function following activation [38]. FoxP3 deficiency in humans leads to a severe autoimmune disease named IPEX and represented by immune dysregulation, polyendocrinopathy, enteropathy, and X-linked syndrome that occur early in infancy [39]. Altogether, it seems that FoxP3 cannot also be a bona fide marker for very specific identification of nTregs in humans.

More recently, it is suggested that conserved noncoding DNA sequence (CNS) elements at the FoxP3 locus, including CNS1-3, encode information defining the size, composition, and stability of the Treg cell population. It is reported that CNS3, the pioneer element which robustly increases the frequency of Tregs generated in the thymus and the periphery, binds to c-Rel, a member of the nuclear factor-*κ*B (NF-*κ*B) family of transcription factors. In contrast, CNS1 is not required for nTreg differentiation but has a critical role in iTreg generation. CNS2 is pivotal for FoxP3 expression in the progeny of dividing Tregs [40]. Moreover, it is demonstrated that NF-*κ*B signaling pathway is a key modulator of FoxP3 expression during nTreg development. It is showed that NF-*κ*B activity through a constitutive active inhibitor of  *κ*B kinase *β* (IKK*β*) transgene in T cells leads to increased number of FoxP3^+^ cells in the thymus and can rescue FoxP3 expression in thymocytes deficient in other pleiotropic signaling molecules [41]. Additionally, it is proposed that c-Rel may act as a pioneer transcription factor in initiating FoxP3 transcription in Treg precursors in the thymus. It is showed that c-Rel modulates FoxP3 transcription directly by binding to cis-regulatory elements at the FoxP3 locus following TCR/CD28 stimulation, including the promoter and the CNS element harboring a permissive chromatin status in Treg precursors [42]. 

Recently, low level expression of CD127 (IL-7R*α*) is also proposed as nTreg recognition marker which correlates with FoxP3 expression and suppressive capacity of nTregs. Activated T cells express high levels of CD127 on their surface [43].

Moreover, the new subtype of nTregs is described which is CD4^+^ or CD8^+^ and usually does not express FoxP3. Their main marker is high expression levels of HLA-G molecules. These cells produce a high level of IL-10 and soluble HLA-G and exert their regulatory function through both contact-dependent and independent mechanisms [44].

It is now apparent that costimulatory signals via molecules such as CD80 (B7-1), CD86 (B7-2), CD28, CD40, and IL-2R*β* are very important for development and maintenance of nTregs [45]. It is reported that CD28 deficiency in mice leads to reduction of suppressive capacity of nTregs [46]. Moreover, CD28 can increase the survival of nTregs via enhancing IL-2 secretion from conventional T cells [47]. The role of IL-2 in function and development of nTregs also substantiated in several studies [48]. It has been suggested that nTreg activation may not be required for the gain of maximum capacity of suppression [49]. In contrast, it has been demonstrated that only preactivated nTregs (through their TCR) can suppress proliferation of non-Treg conventional T cells. Additionally, it is noted that antigen specificity of nTreg target conventional T cells is not important, because inhibitory capacity of nTregs is antigen nonspecific [50].

Surprisingly, it is reported that nTregs can transfer suppressing features to conventional T cells in coculture that this transduction is contact independent and mediated by soluble factors such as IL-10 and TGF-*β* [51]. This regulatory phenotype transferring leads to generation of either Tr1 or Th3 phenotype in conventional T cells [52]. Which factor or condition assesses the fate of these conventional T cells to convert to Tr1 or Th3 phenotype? Jonuleit and Schmitt showed that presence of different subsets of nTregs generates different phenotypes of iTregs. They demonstrate that nTregs are divided into two subgroups based on their integrin expression. nTregs that express  *α*4*β*7 integrin convert CD4^+^ T cells to IL-10-producing Tr1 cells, whereas nTregs that express  *α*4*β*1 integrin generate TGF-*β*-producing Th3 cells [9].

## 4. Tr1 Regulatory Cells 

Tr1 cells are IL-10-producing Tregs distinguished from nTregs based on different cytokine secretion profile, antigen responsiveness, suppression mechanisms, and maybe some cellular markers [53]. IL-10 was identified for the first time as a soluble inhibitory factor generated by mice Th2 cells that could prevent activation and cytokine secretion by Th1 cells; hence that was termed cytokine synthesis inhibitory factor (CSIF) in first [54]. It is reported that IL-10 secreted by Tr1 cells is detectable only 4 hr after their activation and its peak is on 12–24 hr after activation [55]. The various roles are reported for IL-10 in regulation of immune system including downregulation of costimulatory molecules, MHCII, modulation of APCs, prevention of inflammatory mediators secretion, inhibition of T-cell cytokine secretion, enhancing the proliferation and cytotoxic function of CD8^+^ T cell, induction of anergy in T cells, and promoting of B-cell differentiation and survival [56]. 

The most prominent sign in recognition of Tr1 cells from other CD4^+^ T cells is their exclusive cytokine secretion profile. Tr1 cells produce a high level of IL-10 and TGF-*β*, intermediate amount of IL-5, and low amount of IL-2 and IFN-*γ* but do not produce IL-4 (IL-10^high^, TGF-*β* ^high^, IL-5^int^, IL-2^low^, IFN-*γ* ^low^, IL-4^−^) [57]. It is showed that following activation of Tr1 cells through their TCR, they can express the normal levels of activation markers including CD28, CD69, CTLA4, CD25, IL-2R*βγ*, CD40 ligand (CD40L), and HLA-DR [58]. Moreover, it appeared that Tr1 cells can express some of chemokine receptors on their surface which normally express on the Th1 or Th2 cells, such as CXCR3, CCR5 (Th1 chemokine receptors), CCR3, CCR4, and CCR8 (Th2 chemokine receptors) [59]. Although receptor of GATA-3 (ROG) is suggested as Tr1 marker, it cannot be as a bona fide marker for identification of Tr1 cells, because it is showed that ROG is also expressed in activated T cells [60].

 Additionally, it is demonstrated that Tr1 cells cannot express FoxP3 constitutively, but following activation, they upregulate FoxP3 expression to levels like those expressed in activated T cells [61]. This observation is correlated with a fact that Tr1 cells do not need to have FoxP3 for exertion of their suppressor function, because it is reported that Tr1 cells can suppress the conventional T cells independently from FoxP3 expression. Moreover, it is showed that Tr1 cells can differentiate from naive T cells of patients with IPEX disease which lack FoxP3 transcription factor [34]. On the other hand, Veldman et al. identified two subpopulations of Tr1 cells where one of them expresses FoxP3 in pemphigus vulgaris disease [62]. In another study, same group showed that inhibition of FoxP3 expression leads to Tr1 conversion to Th2-like phenotype and loss of suppressive function [63].

As mentioned before, Tr1 regulatory cells are member of iTregs that can differentiate from naive CD4^+^ T cells in some conditions. It is reported that Tr1 cells can be generated *in vitro* by continuing TCR stimulation in the presence of high levels of IL-10 [53]. Moreover, it seems that presence of IFN-*α*  is critical for efficient differentiation of Tr1 cells in addition to IL-10, *in vitro* [64]. Molecules such as CD2 and CD46 are other candidates whose signaling can induce Tr1 differentiation. It is demonstrated that signals resulted from CD2 interaction with its ligand (CD58) leading to promotion of Tr1 induction [65]. Investigation of molecular mechanisms of this induction requires more studies in this field. On the other hand, Atkinson and colleagues showed that cosignaling of CD46/CD3 can induce Tr1 cells with capacity of granzyme B expression [66]. It has been also shown that vitamin D3 and dexamethasone can induce Tr1 differentiation through promotion of autocrine IL-10 generation [67]. Immature dendritic cells are other inducers of Tr1 cell differentiation. It is reported that repetitive stimulation of naive CD4^+^ T cells with allogenic immature dendritic cells leads to induction of IL-10-producing Tr1 cells [68]. 

In spite of general agreement that Tr1 cells are member of CD4^+^ T cells, studies identified CD8^+^ Tr1-like cells that generate IL-10. Induction of these cells was IL-10 dependent. It is showed that stimulation of naive CD8^+^ T cells with activated plasmacytoid dendritic cells or myeloid dendritic cells leads to induction of CD8^+^ Tr1-like cells *in vitro*. Moreover, it is demonstrated that suppressive function of these cells is mediated trough IL-10 [69]. 

Although the cell contact-dependent mechanisms were also suggested for suppressive function of Tr1 cells, it is apparent that the major mechanisms of suppression by Tr1 cells is based on contact-independent pathways, particularly via cytokines such as IL-10 and TGF-*β* [70].

Proliferation capacity of Tr1 cells following TCR stimulation is very low. It is reported that use of anti-IL-10 monoclonal antibody can restore Tr1 cells proliferative capacity partially. Thus, it seems that this anergic condition of Tr1 cells may be in owing to autocrine production of IL-10 by Tr1 cells. With using of cytokines such as IL-2 and IL-15, it comes possible to break this anergic phenotype and expand Tr1 cells *in vitro*. Ability of these cytokines to expand Tr1 cells is due to the expression of receptors such as IL-2R*α*, IL-15R*α*, IL-2/IL-15R*β*, and IL-2/IL-15R*γ* in activated Tr1 cells [71].

Roncarolo et al. suggest distinct migratory features between nTregs and Tr1 cells. They also propose that nTregs can be recruited and activated early during an immune response to control its magnitude, whereas Tr1 cells, which are induced following repeated antigen stimulation, may act later to decrease the immune response and to restore and maintain tolerance [58]. 

## 5. Th3 Regulatory Cells

Th3 regulatory cells identified in mice following oral administration of MBP for tolerance induction, for the first time [72]. It is showed that treatment with MBP leads to induction of TGF-*β*-producing Th3 cells which were MBP specific and inhibited EAE [73]. 

Th3 cells are one of Treg subsets that can differentiate from naive CD4^+^ T cells following ingestion of a foreign antigen through the oral route [8]. As mentioned before, Th3 regulatory cells were identified during the field of investigating mechanisms associated with oral tolerance. Different mechanisms of tolerance are induced following oral antigen administration, including active suppression, clonal anergy, and deletion. Low doses lead to active suppression whereas high doses result in anergy and/or deletion. Th3 regulatory cells are a unique T-cell subset, which mainly secretes TGF-*β*, provides help for IgA secretion, and has suppressive features for both Th1 and Th2 cells [73]. It has been shown that Th3 cells are distinct from the Th2 cells, as CD4^+^ TGF-*β*-secreting cells with suppressive properties have been induced from IL-4-deficient animals. *In vitro* differentiation of Th3 cells from T-cell precursors from TCR transgenic mice is promoted by culture with cytokines such as TGF-*β*, IL-4, IL-10, and anti-IL-12 monoclonal antibody. Th3 MBP regulatory clones are structurally identical to Th1 encephalitogenic clones in TCR usage, MHC restriction, and epitope recognition but generate TGF-*β* with various amounts of IL-4 and IL-10. It has been demonstrated that oral antigen induces Th2, Th3, and nTregs cells and latency-associated peptide^+^ (LAP^+^) T cells. Moreover, induction of oral tolerance is promoted by IL-4, IL-10, anti-IL-12, TGF-*β*, cholera toxin B subunit, Flt-3 ligand, and anti-CD40 ligand [74]. Additionally, it has been shown that *in vivo* induction of Th3 cells and low dose oral tolerance is increased by oral ingestion of IL-4. Anti-CD86 but not anti-CD80 inhibits the differentiation of Th3 cells associated with low-dose oral tolerance [74].

There are controversial reports about of FoxP3 expression in Th3 cells. Although some studies detected FoxP3 in Th3 cells, others suggest that Th3 cells do not express this transcription factor [75]. However, there are some evidence suggesting that Th3 cells can express some nTreg molecules such as CD25 and CTLA-4 [76]. Thus, this question remains unresolved until now: whether Th3 regulatory cells are a distinct Treg subset or are the same activated nTregs? However, induction of Th3 cells in mice with complete lack of nTregs in TGF-*β*-dependent fashion strikingly suggest that Th3 cells are distinct subsets of Tregs and are different from nTregs [77]. It seems that TGF-*β* signaling has a major role in Th3 differentiation. TGF-*β* receptor consists of two different proteins, TGF-*β* receptor type I (TGF-*β*RI) and TGF-*β* receptor type II (TGF-*β*RII), which signal through a serine/threonine kinase domain that phosphorylates transcription factor of SMAD. Active TGF-*β*1 binds to the TGF-*β*RII subunit on the cell surface. The binding of TGF-*β*1 induces the assembly of the activated receptor-ligand heteromeric complex, which results in autophosphorylation of the receptor followed by phosphorylation of R-SMAD (receptor-regulated SMAD). Phosphorylated RSMADs form homooligomeric and heterooligomeric complexes with the comediator SMAD (Co-SMAD). These complexes are translocated to the nucleus where they associate with DNA-binding cofactors, transcriptional coactivators (Co-A), and corepressors to regulate transcriptional activity of the target genes. TGF-*β*1 prevents abnormal T-cell activation through the modulation of Ca^2+^-calcineurin signaling in a SMAD3- and SMAD4-independent manner. However, in Tregs, its effects are mediated, at least in part, through SMAD-signaling. Also, SMAD-independent TGF-*β* signaling pathways are identified including rapid activation of Ras-ERK, TAK-MKK4-JNK, TAK-MKK3/6-P38, Rho-Rac-cdc4 MAPK, and PI3 K-Akt pathways occurring following treatment of cells with TGF-*β* [27, 78].

Although it is showed that Th3 regulatory cells are induced in an antigen-specific manner, they exert their suppressive function in an antigen-nonspecific manner [5]. Th3 cells exert their suppressive action via contact-independent mechanisms and primarily through TGF-*β* secretion. Since TGF-*β* has wide range of expression and affects the function of various cell types, Th3 cells may have a major role in many aspects of immune modulation and T-cell homeostasis [9]. However, recognition of precise properties, biology, and function of Th3 regulatory cells needs identification of new specific markers, which by use of them isolate these cells from other CD4^+^ T cells; that requires more studies in this field.

## 6. CD8^+^ Regulatory T Cells

Although CD8^+^ Tregs were identified earlier than other Tregs subsets, information about them are little, until now [79]. Difficulties in their isolation and characterization as well as lack of specific markers on their cell surface result in existence of little data about their properties, biology, and precise mechanisms of suppressive function [80]. Difficulties in CD8^+^ Treg isolation is partially consistent with their low frequency in periphery whereas it is reported that CD8^+^ Tregs constitute relatively small than 1% of peripheral circulation that may be much higher among intestinal epithelial lymphocytes [24]. It has been also reported that CD8^+^ FoxP3^+^ thymocytes in thymus of mice form about 1% of mature thymocytes [24, 81].

Some markers are suggested for distinguishing CD8^+^ Tregs from conventional CD8^+^ T cells such as CD25 (some subsets), CTLA-4, FoxP3 (some subsets), HLA-DR, CD28 (some subsets), LAG-3, CD27, CD38, CD103, CD122, GITR, and CD8*αα*  [82]. With respect to this profile of expressed molecules in CD8 Tregs, it seems that there is not any specific marker for discrimination of CD8 Tregs from conventional CD8 T cells, because the majority of these molecules are expressed also in activated T cells.

Some subsets of CD8^+^ Tregs are suggested. CD28 expression may divide CD8^+^ Tregs into two subgroups. On the other hand, it is showed that CD8^+^CD28^−^ Tregs can divide into two other subgroups represented by different condition of induction. It is demonstrated that type I CD8^+^CD28^−^ Treg cells are induced via stimulation of naive T cells with allogenic APCs whereas type II CD8^+^CD28^−^ Tregs are generated through incubation with monocytes, IL-2, and GM-CSF [83]. Moreover, another CD8^+^ Treg subset exists in thymus of normal individuals that express molecules like nTreg subsets. This subset expresses molecules such as CD8, CD25, FoxP3, CTLA-4, and GITR where the majority of them can express in nTregs. Additionally, the mechanism of suppression exerted by this subset is cell contact dependent like that observed in nTregs; hence this subset is termed natural CD8^+^ Treg [84]. On the other hand, Uss and colleagues generated the adaptive form of natural CD8^+^ Tregs *in vitro*. They have demonstrated that continuing antigen stimulation of CD8^+^CD25^−^ T cells in the presence of monocytes leads to generation of highly suppressive adaptive CD8^+^CD25^+^FoxP3^+^ T cells *in vitro* [85]. Moreover, it has been also shown that in peripheral blood of normal individuals exists CD8^+^ Tregs with expression of CD103 integrin [86].

It seems that costimulatory molecule CD137 (4-1BB) and IFN-*γ* are important factors in differentiation, induction, and function of CD8^+^ Tregs. Myers and colleagues demonstrated that immunization with ovalbumin (OVA) in combination with anti-4-1BB and polyI: C leads to generation of CD8^+^ Tregs which highly suppressed CD4^+^ T cells. They showed that IFN-*γ* is required for exertion of suppressive function of CD8^+^ Tregs, because IFN-*γ* attaches to CD8^+^ Tregs and leads to secretion of TGF-*β* by CD8^+^ Tregs which in turn suppresses CD4^+^ T cells [87]. It is suggested that TCR stimulation also can lead to induction of CD8^+^ Tregs. Stimulation of peripheral blood mononuclear cells (PBMCs) with anti-CD3 leads to generation of CD8^+^CD25^+^FoxP3^+^ *in vitro* which can potently suppress CD4^+^ T cells [88].

Interestingly, Cone et al. describe CD8^+^ Tregs that are not restricted to MHC class IA, but restricted to Qa-1 molecule which is MHC class IB molecule. Qa-1 generally expresses in some cells such as activated T cells, dendritic cells, and B cells. It has been also demonstrated that Qa-1 can act as a ligand for NKG2A, the inhibitory receptor on NK cells. Therefore, Qa-1 can protect T cells from lyses by NK cells and can mediate inhibitory function of CD8^+^ Tregs into activated T cells [89]. 

It is a little known about suppression mechanisms exerted by CD8^+^ Tregs, and recognizing details in this field requires more studies in the future. However, it is suggested that CD8^+^ Tregs suppress target cells by both contact-dependent and contact-independent mechanisms [90].

## 7. NKT Regulatory Cells

Although the term of natural killer T cell (NKT cell) was described in 1995 for the first time, the first signs of NKT cell existence in mice have been reported in 1987. Three reports showed that there are distinct subsets of  *αβ*TCR-expressing T cells with higher frequency of V*β*8 expression than other conventional T cells [91]. The major cause for this naming of these cells is that these cells share the features from both T and NK cells in their surface markers and immunologic biology. NKT cells coexpress markers such as NK1.1 (CD161) and IL-2R*β* (CD122) that usually express in NK cells and semi-invariant  *αβ*TCR which is specific for T cells [92]. The major features shared by NKT cells are heavily biased TCR gene usage, CD1 restriction, and secretion of high levels of cytokines of Th1 and Th2 phenotypes such as IFN-*γ* and IL-4 [93].

Although NKT cells show some features different from conventional Treg cells, they share some similarities with Tregs that let us classify them as a subtypes of Tregs, in here. The key difference between Tregs and NKT cells is the CD1 restriction in NKT cells and in contrast MHCII restriction in Tregs. NKT cells recognize the glycolipid antigens presented on CD1d molecules, whereas Tregs only recognize the peptides presented on MHCII molecules. It is demonstrated that glycolipids such as  *α*-galactosylceramide (*α*-GalCer) isolated from a marine sponge, isoglobotrihexosylceramide (iGb3), and various bacterial glycosphingolipids have the NKT cell activation potential [94]. It should be noted that humans lack the functional iGb3 synthase enzyme; hence they cannot produce this glycolipid [95]. Tregs have also a diverse TCR repertoire, but NKT cells have a confined TCR repertoire. It is demonstrated that most NKT cells in mice express the invariant V*α*14J*α*18 TCR*α*  chain paired with V*β*8.2, V*β*2, or V*β*7 chains. On the other hand, most human NKT cells express V*α*24J*α*18 with V*β*11. Owing to limited diversity of this population of NKT cells, these cells are named iNKT cells. In spite of these differences between Tregs and NKT cells, they share some similar features. It has been shown that NKT cells like the Tregs can suppress proliferation and cytokine secretion in Th1 and CD8^+^ T cells [96]. Owing to sharing these suppressive functions and declining number and function of NKT cells (like the Tregs) in some autoimmune diseases, we think that NKT cells can also be surveyed as a subtype of Tregs. 

In general, NKT cells are divided into three subtypes based on their reactivity to the glycolipid  *α*-GalCer, TCR  *α*-chains, and CD1-dependency. Type I NKT cells that also named iNKT cells have a limited TCR-*α*  chain repertoire and react to  *α*-GalCer in a CD1d-dependent fashion. Type II NKT cells have a diverse TCR-*α*  chain repertoire, do not react to  *α*-GalCer, but are CD1d dependent. It is noted that type II NKT cells are enriched for cells that have a TCR*α*  chain composed of V*α*3J*α*9 or V*α*8 in combination with V*β*8.2. Interestingly, it has been demonstrated that these cells might also contain a fraction of some *γδ*T cells [93]. However, there are controversial discussions on *γδ*NKT cells because they are NK1.1^+^ but they have no CD1d restriction [97]. Type III NKT cells do not react to  *α*-GalCer and are CD1d independent but have a diverse TCR-*α*  chain. Another classification is based on their CD4 and CD8 surface expression. Most of NKT cells are CD4^+^, and majority of remained cells are double negative CD4^−^CD8^−^ NKT cells (DN NKT). There are also a small number of NKT cells in human (but not in mice) that express CD8 and are CD8^+^ NKT cells [98, 99].

As mentioned before, NKT cells can recognize only antigens presented by CD1d molecules. It is showed that various cell types such as double positive thymocytes, B cells, MQs, dendritic cells, and hepatocytes express CD1d molecule, but it is not understood the precise role of these cells in antigen processing and presentation to NKT cells. However, it is apparent that CD1d molecules are assembled with *β*2-microglobulin in the endoplasmic reticulum (ER) and loaded with ER resident lipid antigens with assistance of lipid transfer proteins such as microsomal transfer protein (MTP). These lipid-CD1d complexes transport to cell surface and subsequently internalize via the endosomal-lysosomal pathway, mediated through cell coating adaptor proteins including adaptor protein 2 (AP2) and AP3 to lysosomes. Once delivered to late endocytic and lysosomal compartments, some complex lipids require processing by resident lipases and glycosidases, such as  *α*-galactosidase A, mannosidase, and hexosaminidase. In late endosomal compartments, ER lipids are replaced with glycolipids, which can be endogenously derived or can be acquired from exogenous sources with assistance of glycolipid containing lipoproteins such as apolipoprotein E and taken up by cells via lipoprotein receptors such as low-density lipoprotein receptor (LDLR), DC-SIGN (CD209), and langerin. In lysosome, lipid transfer proteins such as saposins, GM2-activator protein, and Niemann-Pick's C2 protein (NCP2) facilitate the replacing of ER-derived lipids. Glycolipid-loaded CD1d molecules are then recycled back to the cell surface [99–101]. 

Frequency of NKT cells in various tissues is very different. It is demonstrated that most fractions of NKT cells population are present in liver of both mouse and human. However, the frequency of NKT cells in other tissues such as bone marrow, spleen, thymus, blood, lymph node, and lung is detected, and it is demonstrated that they present in liver with significant higher levels in comparison with other tissues [102]. 

 It is demonstrated that CD4^+^ NKT cells can produce cytokines from both Th1 (IFN-*γ*) and Th2 (IL-4) types whereas DN NKT cells secrete the Th1 phenotype cytokines in human. On the other hand, it is showed that both CD4^+^ and DN NKT cells in mice can secrete the Th2 phenotype cytokine IL-4 [103]. It has been also demonstrated that iNKT cells can produce cytokines such as IL-2, IL-3, IL-5, IL-13, IL-17, IL-21, GM-CSF, and osteopontin following TCR engagement in mice [104, 105].

Since years ago, it is thought that the origin of NKT cells is thymus independent and their appearance was before conventional T cells. It is now known that NKT cells originate from thymus and their appearance in thymus was few later than conventional T cells. It seems that segregation of NKT cells from T-cell lineage occurs at double positive thymocyte stage in the thymus cortex [106]. Although it is showed that origination of NKT cells is thymus dependent, the peripheral maintenance of NKT cells is thymus independent and is attributed to bone marrow [107]. It is demonstrated that NKT cell selection is dependent on recognition of glycolipid antigens presented by CD1d molecules on double positive thymocytes and to lesser extent by other hematopoietic or even corticoepithelial cells [108, 109]. However, it is suggested that thymocytes can play a role in both positive and negative selection of NKT cells. iGb3 is another candidate for positive selection of NKT cells. On the other hand, it is possible that  *α*-GalCer can be as a negative selection inducer during the NKT cells development [110]. It has been also suggested that dendritic cells can induce the negative selection of NKT cells [99].

Although the intracellular signaling pathways that regulate NKT cell development are not fully described, it is known that three major signaling pathways are involved in this field including SLAM-SAP-FYN pathway, NF-*κ*B pathway (following TCR stimulation), and IL-15 pathway [111]. It is showed that mutation of SLAM-associated protein (SAP) leads to X-linked lymphoproliferative syndrome (XLP) that is associated with significant decrease in NKT cell frequency. It is known that SAP can bind to SLAM family receptors such as 2B4, SLAM, CD84, Ly9, and NTB-A and recruits FYN. The formed complex (SLAM-SAP-FYN) opens two signaling pathways: (1) activation of NF-*κ*B via PKC*θ* and (2) inhibition of MAPK that leads to prevention of TCR signaling. Importance of SAP was also demonstrated in mice lacking SAP that results in complete prevention of NKT cell development [112]. It has been also shown that IL-15 and IL-7 derived from thymic stromal cells play a key role in development and function of NKT cells [113]. Lymphotoxin and GM-CSF are other important factors in development of NKT cells [114]. Additionally, it is suggested that transcription factors such as T-bet, GATA-3, Mef, AP-1, Ror-*γ*, IRF1, RUNX1, and Ets-1 are also important in NKT cell development [115, 116]. 

It is suggested that NKT cells can divide into mature and immature stages based on their surface NK1.1 expression. It is demonstrated that immature NKT cells are NK1.1^−^ whereas mature NKT cells can express NK1.1 marker [117]. Although NK1.1^−^ NKT cells are immature in thymus, a significant number of them in other organs might be antigen experienced cells [118]. It has been also suggested that NK1.1^−^ NKT cells are more susceptible to production of Th2-type cytokine IL-4. It is showed that following *in vitro* stimulation of NKT cells, NK1.1^−^ NKT cells secrete higher amount of IL-4 and less amount of IFN-*γ* in comparison with NK1.1^+^ NKT cells [114]. Moreover, NK1.1^−^ NKT cells can produce high levels of cytokines such as IL-10, IL-13, and IL-21 whereas NK1.1^+^ NKT cells express high levels of molecules such as perforin, FasL, and granzyme B [39]. Thus, it might be imaginable that immature NKT cells produce Th2-type cytokines, and this phenotype converts to Th0 phenotype (NKT cells that produce identical amount of both IL-4 and IFN-*γ*) during development process.

## 8. Rheumatoid Arthritis and Regulatory T Cells

Nature has provided the developing immune system with several important checkpoints for the maintenance of tolerance and the prevention of autoimmunity [4]. Tregs functional abnormalities have been identified in different autoimmune diseases, including RA [119]. Tregs have received titanic interest in the past decade, placing them at the centre of immunosuppressive reactions [4] because understanding the development and functions of immunoregulatory cells, the majority of which are CD4^+^ T cells, may elucidate the etiology for loss of self-tolerance [3]. Sempere-Ortells et al. tested the hypothesis that changes in these cells can be linked to the degree of inflammation and relapsing/remission periods. Finally, they reported that the balance status between these cell subsets and their antigen expression would determine the inflammation levels and could thus be linked to the relapsing/remission periods of the disease [120].

Tregs are important cells in the maintenance of immune homeostasis. Defects in Treg function or their reduced numbers have been documented in several human autoimmune diseases, including RA and JIA (juvenile idiopathic arthritis) [121]. The role for Treg cells in RA has been established in both patients and animal models [122]. The high potential of Tregs (in particular natural Tregs), to suppress several arthritic responses both in humans and in animal models of arthritis, makes them therapeutic targets of interest in arthritic conditions such as RA [123]. In addition, increasing insights in understanding the complex mechanisms of action of Tregs have already led to exciting therapeutic advances [2]. 

Here, we review the new current findings of the several subsets of Tregs in RA. So by understanding the role of Tregs in autoimmunity, an effective therapy may be developed to aid in both the treatment and the efficient cure of disease [124].

## 9. Natural Regulatory T Cells and RA

In 1979, Chattopadhyay et al. reported that most of the normal donors had suppressor cell activity in their peripheral blood, whereas a statistically significant number of patients did not have any suppressor cell activity in synovial tissue lymphocytes [125].

RA is a proinflammatory autoimmune disease attributed to failure of both CD4^+^CD25^+^ regulatory T (Tr) and CD8^+^CD28^−^ suppressor T (Ts) cells to control autoreactive CD4^+^CD28^+^ Th1 and autoantibody-producing B cells [126]. CD4^+^CD25^+^ Treg cells can play a critical role in the prevention of autoimmunity, as evidenced by the cataclysmic autoimmune disease that develops in mice and humans lacking the key transcription factor Foxp3 [127]. Mutations in Foxp3 are responsible for the scurfy (sf) mutant mouse and for the autoimmune human diseases including the X-linked fatal “immune dysregulation, polyendocrinopathy, enteropathy, X-linked” (IPEX), autoimmune colitis, and RA [128].

In addition to FoxP3, other molecules have also a role in suppression of RA. For instance, in an *in vivo* murine model, adoptive transfer of Tregs expressing both FoxP3 and Bcl-xL demonstrated more effective suppression of RA than CD4^+^ T cells expressing FoxP3 alone [129]. Moncrieffe et al. suggested that in human CD4^+^ T cells from the inflamed site, CD39 can be highly expressed on two populations, one regulatory Foxp3^+^ CD4 T cells and the other of a memory phenotype [130].

The Foxp3-Tg^+^ (Foxp3 transgenic) CD4^+^CD25^−^ T cells exhibit significantly reduced proliferative response to TCR engagement. Guo et al. reported that Foxp3-Tg mice are resistant to CIA via reduced cellular proliferation of activated T cells [131]. Gonzalez-Rey et al. reported that the human adipose-derived mesenchymal stem cells (hASCs) also stimulate the generation of FoxP3 protein-expressing CD4^+^CD25^+^ Tregs, with the capacity to suppress collagen-specific T-cell responses [132].

Raghavan et al. showed that in synovial tissue, during inflammatory arthritis, FOXP3^+^ cells were present in low numbers within T-cell infiltrates and decreased further after intra-articular glucocorticosteroid administration, in parallel with the general reduction in inflammation [133].

Despite an increased number of Tregs, the persistence of inflammation in the rheumatoid joints suggests that Tregs are unable to suppress ongoing disease [134, 135], perhaps due to an inhibition of their functions by proinflammatory cytokines or because of the increased number of activated effector T cells [134–136]. Thus, paradoxically, RA patients have the elevated numbers of circulating CD4^+^CD25^high^ T cells (composed of CD4^+^CD25^high^FoxP3^+^ Treg cells and activated CD4^+^CD25^high^FoxP3^−^ effector cells); however, the inflammation is still ongoing [135]. In addition, protein kinase C-theta (PKC-theta) which is sequestered away from the Treg immunological synapse inhibits Treg-mediated suppression. Zanin-Zhorov et al. examined and showed that PKC-theta blockade enhances Treg function. Therefore, Treg freed of PKC-theta-mediated inhibition can function in the presence of inflammatory cytokines and thus have therapeutic potential in control of inflammatory diseases [137].

Van Amelsfort et al. suggested that the interaction of CD4^+^, CD25^+^ Treg cells with activated monocytes in the joint might lead to diminished suppressive activity of CD4^+^, CD25^+^ Treg cells *in vivo*, contributing to the chronic inflammation in RA [136]. Meanwhile, in RA, a loss in the immunological self-tolerance causes the activation of autoreactive T cells against joint components and subsequent chronic inflammation [132]. Chen et al. showed that vasoactive intestinal peptide (VIP) had a great protective effect on chicken collagen II-induced experimental arthritis (CIA) model. Disease suppression was associated with the inhibition of T-cell proliferation, shifting the immune response toward a Th2-type response and expanded CD4^+^CD25^+^ Treg in the periphery, which inhibited autoreactive T-cell activation/expansion [138].

Heo et al. reported that IL-10 induces Foxp3^+^ Tregs in the human CD4^+^ T-cell population and on the other hand, IL-17 is overexpressed in autoimmune disease patients, whereas IL-10 suppresses IL-17 expression. It has been reported that IL-10 is useful in the treatment of autoimmune diseases [139]. IL-15 expression of Fibroblast-like cells from the synovium of RA patients (RASFib) exerts a dual action on the equilibrium between CD4^+^CD25^+^ Treg and CD4^+^CD25^−^ responder T cells (Tresp) by potentiating the suppressive effect of Treg while augmenting the proinflammatory action of Tresp; the result is a shift of the Treg/Tresp balance toward a proinflammatory state [140]. Additionally, Treg with superior suppressive potency were present in the peripheral blood and the synovial fluid of RA patients, but this enhanced immunoregulatory activity was not able to overcome the increased secretion of pathogenic cytokines by RA-Tresp, indicating that RA patients demonstrate an altered Treg/Tresp equilibrium *in vivo* [13, 140]. The expanded Treg cells with enhanced biological function may provide an opportunity to restore the proper balance of immunity and tolerance, suggesting the potential of using Treg cell therapy for treatment of immunomediated diseases [141].

The TNF-*α*  plays a central role in RA and current biotherapies targeting TNF-*α*  are effective in RA treatment [142]. A number of CD4^+^, CD25^++^ T cells which is expressed membrane-bound TNF-*α*  displayed reduced anti-inflammatory cytokine production and less potent suppressor capacity [143]. Thus, we can restore the suppressive capacity of CD4^+^, CD25^++^ T cells by anti-TNF-*α*  therapy in RA patients [143]. Treatment of RA with anti-TNF-*α*  monoclonal antibodies such as infliximab and adalimumab has been found to induce and restore the functions of Tregs [134].

CIA is an established mouse model of disease with hallmarks of clinical RA. Saouaf et al. investigated the effects of HDACi (Histone/protein deacetylase inhibitors) therapy on regulatory T-cell function in the CIA model and valproic acid (VPA) treatment on both the suppressive function of CD4^+^CD25^+^ Tregs and the numbers of CD25^+^FOXP3^+^ Tregs *in vivo*. Finally, they reported that the administration of HDACi, VPA, significantly decreased disease incidence and severity in CIA [144]. Whereas there is a hypothesis that Treg defect in RA is linked with abnormalities in the expression and function of CTLA-4. Wei et al. speculated that a decreased frequency of CD4^+^CD25^high^ Tregs and lower level of CTLA-4 (cytotoxic T-lymphocyte-associated protein 4) expression on CD4^+^CD25^+^ Tregs might play a role in the immunoregulation of JIA [119, 145]. Flores-Borja et al. reported that regulation of T-cell receptor signaling by CTLA-4 is impaired in RA Treg and associated with delayed recruitment of CTLA-4 to the immunological synapse. Thus, artificial induction of CTLA-4 expression on RA Treg restores their suppressive capacity [119].

In another study, the frequencies of CD4^+^CD25^high^ Tregs were significantly higher in the third trimester compared to 8 weeks postpartum in patients with RA and controls. Then numbers of CD4^+^CD25^high^ Tregs inversely correlated with disease activity in the third trimester and postpartum. Therefore, the related quantitative and qualitative changes of Treg in pregnancy suggest a beneficial effect of Treg on disease activity [146]. In addition as females have a higher incidence of autoimmune diseases and Tregs play a crucial role in preventing autoimmunity, it might be logical to hypothesize that the females might have a lower number of Treg as compared to males. The data presented by G. Afshan et al. in 2012 showing that there is a lower regulatory T-cell percentage in females than in males could be one of the reasons for increased predisposition of females to autoimmune diseases [147]. Also, Zaiss et al. showed that increasing Treg cell numbers improved clinical signs of arthritis and suppressed local and systemic bone destruction. Thus, enhancing the activity of Treg cells would be beneficial for the treatment of inflammation-induced bone loss observed in RA [148].

Xinqiang et al. demonstrated that a single *i.v.* injection with novel tolerizing DNA vaccine pcDNA-CCOL2A1 (chicken type II collagen) can induce a potent immune tolerance against CIA. The action mechanism behind this efficacy can be at least partially attributed to increased CD4^+^CD25^+^ T regulatory cells. The pcDNA-CCOL2A1 alone seems to be as effective as the current “golden standard” treatment, methotrexate [149]. In a novel study by Yakimchuk et al. in 2012, keratinocyte growth factor (KGF) showed therapeutic effect on CIA depending on immunoregulatory mechanisms, suggesting that KGF treatment leads both to an outflow of naive T cells from the thymus and to a statistically significant increase in the percentage of CD4(+)Foxp3(+) T(regs) in the periphery [150].

## 10. Tr1 and Th3 Regulatory T Cells and RA

Another important category of immunosuppressive cells consists of conditionally induced Tregs such as Tr1, Th3, and various other CD4^+^ lymphocytes [3]. In addition, these subsets of Treg cells, type 1 T regulatory (Tr1) and Th3 cells, exert their suppressive capacity via cytokines such as interleukin-10 and TGF-*β* and are contact independent [151].

Appel et al. reported that there is a different cytokine secretion pattern in the synovial membrane of reactive arthritis (ReA) and RA. For T cells in ReA, they found a typical cytokine secretion profile for T regulatory cells 1 (Tr1), with an elevated level of IL-10- and TGF-*β*-secreting cells [152]. In addition, as mentioned previously, Gonzalez-Rey et al. investigated the effects of human adipose-derived mesenchymal stem cells (hASCs) on human collagen-reactive RA T-cell proliferation and cytokine production. They showed that the numbers of IL10-producing T cells and monocytes were significantly augmented upon hASC treatment [132].

On the other hand, The *Escherichia coli* bacterial extract (OM-89), which induced a strong production of IL-10, is used in the treatment of RA. Toussirot et al. reported that OM-89 has immunomodulatory properties by inducing changes in PBMC cytokines release suggesting an induced Tr1 response to OM-89 [153].

Moreover, Kavousanaki et al. suggested that mature plasmacytoid dendritic cells (DCs) from RA patients with low disease activity, but not those from healthy controls, expressed high levels of indoleamine 2,3-dioxygenase and promoted the differentiation of allogeneic naive CD4^+^CD25^−^ T cells into interleukin-10-secreting Treg cells, or Tr1 cells, which showed poor proliferation *in vitro*. These plasmacytoid DC-primed Treg cells potently suppressed the proliferation of autologous naive CD4^+^ T cells, in a dose-dependent manner. Therefore, modulation of the immune response by plasmacytoid DCs might provide the novel immune-based therapies in autoimmunity and transplantation [154].

Immunotherapy of RA using oral-dosed native chicken or bovine type II collagen (nCII) to induce specific immune tolerance is an attractive strategy. Xi et al. described a novel recombinant peptide rcCTE1-2 which contains only two tolerogenic epitopes (CTE1 and CTE2) of chicken type II collagen (cCII). Furthermore, 50 *μ*g/kg/d of rcCTE1-2 could lower the level of anti-nCII antibody in the serum of CIA animals, decrease Th1-cytokine INF-*γ* level, and increase Th3-cytokine TGF-*β*(1) produced by spleen cells from CIA mice after *in vivo* stimulation with ncCII [155].

In another study, Xue et al. showed that their novel targeted DNA vaccine encoding *Pseudomonas* exotoxin A and costimulatory molecule B7-2 without autoantigens induced a shift from Th1 to Th2 and Th3 cellular and cytokine profiles and a decrease in CD4^+^/CD8^+^ cell ratios in a CIA model [126].

Xinqiang et al. suggested that the mechanism underlying the therapeutic efficacy of low-dose methotrexate, the current “gold standard” treatment in experimental RA, could be at least partially attributed to the increased production of CD4^+^CD25^+^ Treg cells. These cells induced a Th1-to-Th2 shift, downregulated Th1 cytokines, and upregulated both Th2 and Th3 cytokines [126, 156]. Furthermore, as mentioned previously, Xinqiang et al. investigated the characterization of a novel DNA vaccine that is a potent antigen-specific tolerizing therapy (the novel tolerizing DNA vaccine pcDNA-CCOL2A1). The action mechanism behind this efficacy can be at least partially attributed to increased CD4^+^CD25^+^ Treg cells, which specifically downmodulate the T lymphocyte proliferative response to CCII, induce a shift of Th1 to Th2 cells, and downregulate Th1-cytokine TNF-*α*, while upregulating both Th2-cytokine (IL-10) and Th3-cytokine (TGF-*β*) [149].

## 11. CD8^+^ Regulatory T Cells and RA

Essentially, disease-specific approaches may be necessary to identify CD8^+^ Ts optimally suited to treat immune dysfunctions in different autoimmune syndromes [157]. CD8 T suppressor (Ts) cells may directly inhibit other T cells or condition antigen-presenting cells in such a way that immune amplification steps are dampened [158]. As regulatory/suppressor T cells can suppress immunity against any antigen, including self-antigens, they emerge as an ideal therapeutic target. Several distinct subtypes of CD8^+^ Ts have been described that could find application in treating RA or SLE (systemic lupus erythematosus) [157].

For therapeutic aims, CD8 Ts cells could either be generated *in vitro* and transferred into the host or their numbers and activity could be modulated by treating the patient with established or novel immunomodulators [158]. In a xenograft model of human synovium, Suzuki et al. reported that adoptively transferred CD8^+^ T cells characterized by IL-16 secretion have also exhibited disease-inhibitory effects. In mice with polyarthritis, CD8^+^ Ts suppressed inflammation by IFN-*γ*-mediated modulation of the tryptophan metabolism in APC [157].

In RA, patients with active inflammation had an increased percentage of IL-4^+^ CD8^+^ T cells. Higher frequencies of IL-4^+^ CD8^+^ T cells were also found in CD8^+^ T-cell lines from patients with arthritis. Interestingly, most IL-4^+^ CD8^+^ T cells produced TNF-*α*  [159]. Expansion of IL-4^+^ CD8^+^ T cells, which may include precursors of a regulatory CD8^+^ T-cell subset, may represent a general response to chronic joint inflammation [159].

Pawlowska et al. measured the distribution of CD4^+^ and CD8^+^ T cells, regarding CD28 expression, both in peripheral blood (PB) and synovial membrane (SM) of RA and osteoarthritis (OA) patients, on the same day. Their study demonstrated the hypothesis that OA may also (like RA) be a disease with a local immunological involvement. They reported that older OA subjects were also characterized by higher values of the SM/blood ratio of both CD4^+^CD28^+^ and CD8^+^CD28^+^ subpopulations than RA or younger OA patients [160]. In another study Prelog et al. reported that the total counts of CD8^+^CD28^+^ and CD8^+^CD28^+^CD45RA^+^ T cells were inversely correlated to chronological age in JIA patients and healthy donors (HDs). In JIA patients, percentages of CD8^+^CD28^+^CD45RA^+^ T cells and of CD62L-expressing CD8^+^CD28^+^CD45RA^+^ T cells showed a negative correlation with age [161].

Baek et al. tested the hypothesis that the differential expression and function of chemokine and/or adhesion molecules explain why CD4^+^ T cells accumulate within perivascular cuffs, whereas CD8^+^ T cells distribute diffusely within the tissue. Finally, they showed that the synovial fluid (SF) CD8^+^ T cells were much less promiscuous in their expression of chemokine receptors than SF CD4^+^ T cells, so that the alpha (6)*β* (1) integrin was highly expressed on PB CD4^+^ T cells, but not on PB CD8^+^ T cells [162].

## 12. NKT Regulatory Cells and RA

iNKT cells are a distinctive subtype of CD1d-restricted T cells, which recognize glycolipid antigens presented by the CD1d molecule, involved in regulating autoimmunity and capable of producing various Th1, Th2, and Th17 cytokines [163, 164]. These findings suggest that iNKT cells are activated early in the course of CIA and contribute to the pathogenesis of arthritis. Therefore, iNKT-cell activation may be a valid treatment target in RA. Moreover, the clinical and histological signs of arthritis were improved by the functional blockade of iNKT cells by a monoclonal antibody to CD1d at the early phase of the disease [163]. Segawa et al. suggested that the low plasma levels of soluble CD1d (sCD1d) protein in RA patients reduce the number and thus activation of peripheral NKT cells (IFN-*γ* production) [164].

Data from rodent models show that iNKT cells are key regulators of many immune responses including autoimmune arthritis, but their role in human diseases is unclear. iNKT cell deficiency is present in patients with RA and other inflammatory arthropathy. Normal iNKT cell frequency predicts noninflammatory causes of joint pain [165]. In comparison with healthy controls, RA patients had a decreased frequency of peripheral blood iNKT cells. Moreover, the proliferative response of this subset to  *α*-galactosylceramide was also diminished in the patient group [165]. Activation of iNKT cells by their exogenous ligand alpha-galactosylceramide (alpha-GalCer) exerts therapeutic effects in autoimmune diseases such as RA [163]. Furthermore, iNKT cell frequency correlated inversely with the systemic inflammatory marker, C-reactive protein [165]. 

Biomarkers of clinical response to rituximab (RTX) therapy (anti-CD20 therapy) and early predictors of outcome are still under investigation [166]. Parietti et al. demonstrated that the number of iNKT cells is altered in RA patients and that following rituximab therapy, clinical remission of RA is associated with an increase of iNKT cell frequency [163]. Increased frequency of the peripheral regulatory CD4^+^CD25^high^ T-cell subset and the CD3^−^CD16^−^CD56^bright^ NK cell subset after RTX therapy were also observed in all patients. In addition, an increased population of NKT cell subsets was observed in the patients with clinical response [166].

## 13. Conclusions

However, the role of autoreactive CD4^+^ T cells particularly Th1, Th17, and somewhat regulatory T cells in pathophysiology of RA is reliable, but data in CD8^+^ T cells is variable. For instance, several animal studies suggest that these cells may have predominantly a proinflammatory effect in disease process, whereas other studies claim that they have mainly a regulatory role in inflammatory joints and could be a subset of Tregs [167]. Moreover it seems that functional blockades of Tregs play a crucial role in immunopathogenesis of RA and CIA perhaps due to an inhibition of their functions by proinflammatory cytokines or because of the increased number of activated effector T cells or perhaps due to the fact that some fully differentiated Tregs may be unstable [134–136]. With this objective, increasing Treg cell numbers as a therapeutic strategy was performed by Zaiss et al. and showed that enhancing the activity and increasing Treg cell numbers would be beneficial for the treatment of inflammation-induced bone loss observed in RA and improved clinical signs of arthritis [148]. The ability of Treg subsets in particular nTregs, to suppress several arthritic responses both in humans and in animal models of arthritis, made them therapeutic targets of interest in RA. However, in theoretical perspective and some studies in animal model, *in vitro* generation and transfer of Tregs into the RA patient may be beneficial, but the use of this method in human disease is remarkably scarce because on one hand some Tregs subsets are able to transdifferentiate *in vivo* into effector memory T cells that secrete inflammatory cytokines. On the other hand general immunosuppression and increased susceptibility to infection are followed by application of polyclonal Treg therapy. Therefore the use of monoclonal Tregs might be recommended but needs further investigation in human RA.
